# Foreign Bodies in the Stomach

**Published:** 1880-12

**Authors:** Chas. L. Dayton


### FOREIGN BODIES IN THE STOMACH.

REPORTED BY CHAS. L. DAYTON, M. D.

THE following case which came under my observation many years ago is exceptional, and of sufficient professional interest to be reported. It demonstrates that in gastric diseases there is great difficulty in forming a correct diagnosis, and also in reaching a reliable prognosis, the problem only yielding a satisfactory solution through a post-mortem examination.

Mr. S., aged 45, residing at Black Rock, for a period of six months, had complained of gastric pain with nausea, and other symptoms of indigestion; he presented the appearance of one suffering with scirrhus of the stomach, or aggravated dyspepsia. Failing to secure relief after consulting several physicians, he consented to accompany me with a view to consult Prof. Austin Flint, senior, at that time residing in Buffalo. Prof. F. examined the patient thoroughly, and expressed the opinion that he would ultimately recover. Two days afterward the patient suddenly died. At the autopsy, in the presence of Drs. L. P. Dayton, Tobie and Beaman, the stomach was removed. It contained a tumblerful of prune pits; the pyloric orifice was so far occluded by the induration of the surrounding tissues that it admitted only the passage of a small catheter. About three inches from the pyloric orifice the stomach was perforated, probably through the influence of the prunes. His wife stated that he had not eaten prunes in five or six months, and could offer no explanation for his swallowing the pits.

The case is interesting on account of the presence of so large a quantity of foreign substances in the stomach, of the similarity of symptoms to those usually occurring in ulceration and scirrhus, and of the obscurity often attending gastric and intestinal disease which is cleared up only through the post-mortem examination.

